# Can We Infer Inter-Individual Differences in Risk-Taking From Behavioral Tasks?

**DOI:** 10.3389/fpsyg.2018.02307

**Published:** 2018-11-21

**Authors:** Stefano Palminteri, Coralie Chevallier

**Affiliations:** ^1^Laboratoire de Neurosciences Cognitives, Institut National de la Santé et de la Recherche Médicale, Paris, France; ^2^Département d’Etudes Cognitives, Ecole Normale Supérieure, Paris, France; ^3^Institut d’Etudes de la Cognition, Université de Paris Sciences et Lettres, Paris, France

**Keywords:** risk-taking, inter-individual variability, behavioral phenotype, behavioral economics, correlational psychology

## Abstract

Investigating the bases of inter-individual differences in risk-taking is necessary to refine our cognitive and neural models of decision-making and to ultimately counter risky behaviors in real-life policy settings. However, recent evidence suggests that behavioral tasks fare poorly compared to standard questionnaires to measure individual differences in risk-taking. Crucially, using model-based measures of risk taking does not seem to improve reliability. Here, we put forward two possible – not mutually exclusive – explanations for these results and suggest future avenues of research to improve the assessment of inter-individual differences in risk-taking by combining repeated online testing and mechanistic computational models.

## Introduction

In a recent series of studies Frey et al. (2017) investigated the relationship between different measures of risk-sensitivity in a laboratory-based experiment involving over a thousand participants (N∼1500) (Frey et al., 2017; Pedroni et al., 2017). By comparing standard behavioral tasks, personality questionnaires, and reports of actual frequency of risky behaviors, the authors were able to demonstrate that behavioral tasks are consistently less reliable than questionnaires. First, performance in risk-taking tasks were less correlated to actual frequency of risky behaviors than personality scores, which suggests that external validity is low. Second, behavioral measures were less correlated among themselves than personality scores and frequency measures, which suggests that they tap constructs that are less consistent (low between-task reliability). These findings are not isolated: other studies from other laboratories, involving smaller number of subjects and behavioral tasks, reached very similar conclusions (Corsetto and Filippin, 2013; Attanasi et al., 2018). Beyond raw behavioral measures, a computational modeling approach using cumulative prospect theory (CPT) parameters (decreasing marginal utility, loss aversion and subjective weighting of probabilities) failed to improve between-task reliability. Finally, test-retest reliability was lower for behavioral tasks than for personality scores. Strikingly, preliminary evidence suggests that these issues generalize to many behavioral tasks beside decision-making under risk, such as reinforcement learning (Enkavi et al., 2018). These findings are not isolated: other studies from other laboratories, involving smaller number of subjects and behavioral tasks, reached very similar conclusions (Corsetto and Filippin, 2013; Attanasi et al., 2018).

Low external validity and-reliability is extremely worrying for the development of behavioral economics applications and (by extension) for the neuroeconomics research framework, where risk preferences are commonly assessed and elicited using behavioral tasks. In addition, the unreliability of behavioral measures is also problematic for the computational psychiatry research framework that has recently placed great emphasis on the use of cognitive and behavioral phenotyping tools. The idea behind these frameworks is that behavioral measures can be used to phenotype patients at the individual level and ultimately work as tools to perfect diagnosis, personalize care, and assess the efficacy of new treatments or drugs in clinical trials (Huys et al., 2016). In this context, it is therefore vital that behavioral tasks generate results that are stable and predictive of real life outcomes.

In addition to questioning approaches based on behavioral phenotyping tools, these findings also raise a profound epistemological challenge. Given that real life frequency of risky behaviors is the reflection of past choices, why then, do personality measures – that are based on *questionnaires* – explain real life behaviors better than behavioral measures – that are based on *choices*? And why would the same subjects produce different choices when presented with the very same task twice?

## Two Possible Explanations

We put forward two possible answers for these puzzling results and fundamental questions (low external validity and consistency of behavioral measures): The first possibility is that these findings reflect a **problem with the instrument;** The other possibility is that these findings reflect a **problem with the construct**.

The “**problem with the instrument**” argument has been explicitly put forward by the authors of the studies (Frey et al., 2017; Pedroni et al., 2017). According to this hypothesis, the low external validity and reliability of the behavioral tasks derive from intrinsic limitations of the tasks. For instance, it has been argued that low between-task consistency between behavioral measures derives from the fact that each task involves both central (risk sensitivity) and peripheral processes (responses, stimuli), whose variability may affect the results. Low test-retest reliability should also be expected given that behavioral and cognitive tasks are traditionally designed to reduce between-subjects variance and to maximize between-conditions variance, such that the very features that make a behavioral task “successful” (high reproducibility of the “average” results) make it unsuited to assess inter-individual differences (Hedge et al., 2017). As nicely summarized by Hedge et al. (2017):

“Experimental effects become well established – and thus those tasks become popular – when between-subject variability is low. However, low between-subject variability causes low reliability for individual differences, destroying replicable correlations with other factors and potentially undermining published conclusions drawn from correlational relationships.”

Propensity measures on the other hand, are designed to maximize inter-individual differences. In addition, a good test-retest reliability is a *condicio sine qua non* for the publication of personality questionnaires, hence their good temporal consistency. Finally, in the context of the specific set of studies at hand, it is also worth noting that the frequency measures were assessed using self-report questionnaires, which involve the same response modality as the personality measures. Furthermore, risk propensity and risk frequency assessments shared similar content and it should come as no surprise that subjects provide similar responses to similar questions, e.g., in order to present a coherent image of themselves (a good “narrative”). In statistical terms, this would result in an artificially increased correlation between frequency and personality measures. Taken together, these features may inflate the consistency and validity of the personality measures. Finally, self-reported questionnaires are well-known for eliciting edulcorated representations that are influenced by a range of social norms (Edwards, 1953). To overcome the issues raised by self-reported frequency of risk behaviors, personality and behavioral measures should be tested against objective assessments of risky behaviors (e.g., expired CO_2_ for smoking, medical records, etc.).

The argument that there is a “problem with the instrument” also applies to the mathematical model used to quantify risk propensity parameters. The authors indeed focused on CPT, which is a widely used descriptive model originally designed to explain one-shot decisions. But three features of CPT may undermine the internal consistency of model-based measures of risk sensitivity (Tversky and Kahneman, 1992). First, different tasks engage different peripheral processes but the same CPT model is applied to various behavioral tasks with no task-specific adjustment of the functional form. Second, and more importantly, CPT parameters are assumed to be static and not affected by the individual’s history of choice, by relevant contextual factors or by feedback. In that respect, CPT is a purely descriptive model rather than a mechanistic model. Third, CPT parameters are often correlated and it is often hard to disentangle their respective contribution to risky behavior using standard fitting procedures. This is in part because different parameter values can produce the same behavioral phenotype (e.g., loss aversion) (Nilsson et al., 2011), which may undermine the power of the model to unambiguously predict particular behavioral profiles.

**FIGURE 1:**
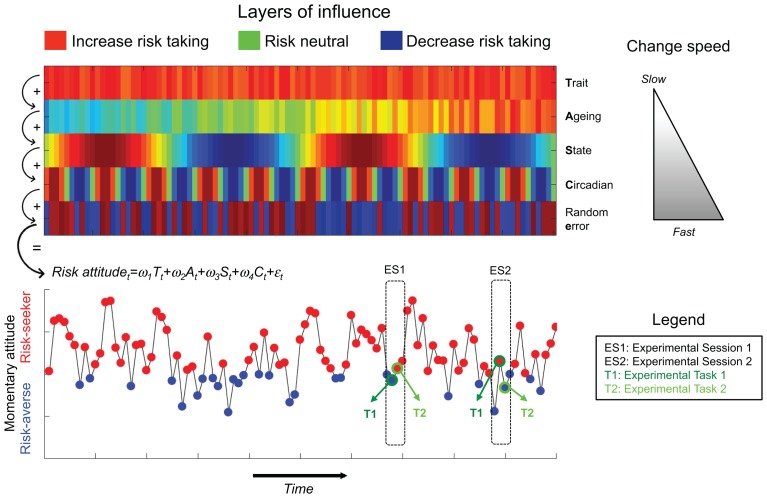
The figure schematizes how low consistency of behavioral measures of risk may arise from the multi-layer model. At the top, we represent the different factors that influence the probability to express a given behavioral phenotype at a given time point in addition to random error. We consider a simplified case in which only two phenotypes are possible: red (risk seeking) and blue (risk aversion). The different layers change at different time constants (as exemplified by the gray triangle on the right). At a given time point (t) the momentary risk attitude is the weighted sum of the different layers of influence plus random error. A given subject is tested in two experimental sessions (ES1, and ES2) with two behavioral tasks supposed to measure the same behavioral phenotype (T1 and T2). The multi-layer model may explain why behavioral measures are not consistent between-tasks and between-sessions.

The “**problem with the construct**” argument implies that behavioral tasks provide a genuine estimate of the subject’s momentary risk attitude at the time of testing, but that risk attitude itself changes over time. This is plausible if we assume that momentary risk attitude is influenced by multiple factors. To illustrate this idea, we now consider a simplified case involving two possible phenotypes, a risk-seeking phenotype (red) and a risk-averse phenotype (blue), and we propose a multi-layer model in which momentary risk attitude corresponds to the weighted sum of different sources of influence that change with different time constants (Figure 1). In this toy example, the first layer corresponds to the subject’s “trait,” which is determined by her genotype and which remains stable over her lifespan. The last layer corresponds to random (or unpredictable) factors, such as unexpected external stimuli and contextual factors. In between these two extremes, we hypothesize that additional sources of influence are at play, such as very slow age-related changes and very fast circadian rhythms. According to this model, a subject tested twice with the same behavioral task at different time points will not necessarily display the same phenotype. Within this framework, the fact that propensity measures produce more stable results can be explained by the fact that filling out questionnaires relies on cognitive processes that do not involve risk attitude *per se*, such as robust averaging of previous experiences stored in episodic memory or introspection.

Crucially, there is evidence demonstrating that these various layers are indeed relevant to understanding decision-making under risk: genetic factors influence risk-related behaviors (Linner et al., 2018), behavioral measures of risk sensitivity evolve across the life-span (Weller et al., 2011), and are affected by hormonal and circadian factors (Lazzaro et al., 2016; Glimcher and Tymula, 2017), mood states (Stanton et al., 2014), as well as momentary arousal (FeldmanHall et al., 2016). Importantly, the same factors are involved in other decision-making processes such as cooperation in economics dilemmas, a field where behavioral tasks also predict real life behaviors poorly (Gurven and Winking, 2008; Winking and Mizer, 2013). By contrast, propensity measures, as implemented by questionnaires, are designed to assess participants’ prototypical behavior averaged across long period of times, thus canceling out momentary trends. In other words, questionnaires are designed to assess stable “*traits*.” In many cases, participants are explicitly instructed to extract their prototypical behavior with formulations such as “*describe yourself as you generally are*” and to ignore the variability induced by circadian or age-related changes.

## Conclusion and Perspectives

Recent evidence based on large-scale behavioral testing shows that behavioral measures in cognitive tasks are outperformed by propensity measures from personality questionnaires, in terms of external validity (i.e., correlation with frequency measures) and reliability (between-tasks consistency and test-retest reliability). We delineate two possible – not mutually exclusive – interpretations of these results. The pessimistic “problem with the instrument” argument states that behavioral tasks are not suited to investigate inter-individual differences. The optimistic “problem with the construct” argument states that variability in behavioral tasks reflects true changes in momentary risk attitude. According to this view, behavioral tasks reflect true momentary risk attitude and will the quantification of the relative weights of the different layers.

At the moment, personality questionnaires appear to be the best psychological tools to predict the frequency of real-life risky behavior. Should we then, abandon the quest for behavioral measures of individual variability? Probably not. Questionnaires are hugely informative when it comes to providing an accurate description of the variability with which personality traits manifest but they cannot be used to trace back the cognitive and neural mechanisms that together produce such variability. The paucity of robust behavioral tools to characterize inter-individual differences therefore constitutes an important obstacle in building proper models of cognitive variability.

Developing behavioral biomarkers, however, requires a proper re-think in the way cognitive scientists design tasks so that they maximize between-subjects variance. One promising possibility is to shift from fixed and passive designs to **active and adaptive** ones. Adjusting task parameters online could indeed correct for momentary changes in baseline performance that may affect the assessment of risk preferences. These results also highlight the importance of **repeated testing**, which has now become considerably easier with the development of smart-phone based behavioral experiments. Repeated testing should also allow us to test the multi-layer hypothesis, to attribute precise coefficients to the different layers, and by averaging performance over experiments, to infer participants’ trait-level phenotype. The issue related to the ambiguous relationship between CTP parameters and behavioral profiles and their correlation may be solved by implementing principal component analyses instead of working with the raw parameters and by implementing hierarchical model fitting (Nilsson et al., 2011). This approach would of course require external validation to assess which component reflects risk sensitivity but we believe it is a valuable alternative to current methods. Ultimately, developing and refining **mechanistic and dynamic models** of risk preferences that integrate learning processes and contextual factors, might also allow for a better quantification of risk preferences at the individual level. A promising way to design these models could be the development of choice prediction competitions, a method that already commonly used in the machine learning literature (Erev et al., 2017). Even more ambitiously, these prediction competitions would include data collection at multiple time points as well as external validation by real life outcomes.

## Author Contributions

SP designed the review. SP and CC wrote the review.

## Conflict of Interest Statement

The authors declare that the research was conducted in the absence of any commercial or financial relationships that could be construed as a potential conflict of interest.
